# Comparison of Percutaneous Core Needle Biopsy Results in Patients Who Previously Underwent Open and Robot-Assisted Kidney Transplantation

**DOI:** 10.3390/jcm13154518

**Published:** 2024-08-02

**Authors:** Fatih Gokhan Akbay, Zeki Toprak, Mithat Eksi

**Affiliations:** 1 Department of Nephrology, University of Health Sciences, Bakırkoy Dr. Sadi Konuk Education and Research Hospital, Istanbul 34147, Turkey; fatihgokhanakbay@gmail.com; 2 Department of Nephrology, University of Health Sciences, Umraniye Education and Research Hospital, Istanbul 34760, Turkey; 3 Department of Urology, University of Health Sciences, Bakırkoy Dr. Sadi Konuk Education and Research Hospital, Istanbul 34147, Turkey; mithat_eksi@hotmail.com

**Keywords:** robot-assisted surgery, kidney transplantation, needle biopsy

## Abstract

**Objective:** The objective of this study was to investigate the safety and efficacy of percutaneous graft biopsy, specifically in patients who have undergone robotic kidney transplantation, a topic that has received limited attention in the existing literature. While percutaneous graft biopsy is well established in patients who have undergone open transplantation, its application in robotic transplantation remains relatively unexplored. **Material and Methods:** A retrospective analysis was conducted on patient records spanning from 2013 to 2024, focusing on those who underwent graft biopsy due to acute graft dysfunction. The cohort was bifurcated into two distinct groups: individuals who underwent open kidney transplantation and those who underwent robotic kidney transplantation. **Results:** The study encompassed a total of 89 patients, with 64 having undergone open kidney transplantation and 25 having undergone robot-assisted kidney transplantation. The mean age of the patients was 40.61 (±12.26) years, with 60 (67.4%) being male and 29 (32.6%) being female. Comparative analysis revealed no significant disparities in age, gender distribution, body mass index, donor type (cadaveric versus living), or rates of graft loss between the two groups. Furthermore, examination of the total complication rates did not uncover any noteworthy differences between the cohorts. **Conclusions:** Ultrasound-assisted percutaneous needle biopsy is a reliable method in patients who have undergone robot-assisted kidney transplantation in cases of both indication-based and protocol biopsies. This study underscores the reliability of ultrasound-assisted percutaneous needle biopsy as a viable method for patients who have undergone robot-assisted kidney transplantation. By shedding light on the safety and efficacy of percutaneous graft biopsy in the context of robotic transplantation, this research contributes to the expanding body of knowledge in the field, providing valuable insights for clinical practice and future research endeavors.

## 1. Introduction

Percutaneous core needle biopsy stands as the gold standard for the evaluation and diagnosis of dysfunction in transplanted kidneys [1]. This procedure is widely recognized and thoroughly defined in patients who have undergone open kidney transplantation (OKT). However, there exists a limited body of research in the current medical literature that focuses on the safety and efficacy of this biopsy technique in patients who have undergone robot-assisted kidney transplantation (RAKT).

For patients who have undergone RAKT, the unique anatomical considerations pose a challenge for the standard percutaneous needle biopsy. Specifically, due to the intraperitoneal positioning of the transplanted kidney, the laparoscopy-assisted needle biopsy has been recommended as a safer alternative [2]. This recommendation is based on the premise that the traditional approach might pose higher risks due to the altered anatomical context post-RAKT.

The present study was conducted with the aim of assessing both the safety and efficacy of ultrasound-assisted percutaneous needle biopsy in this specific subset of patients who have undergone RAKT. By focusing on this particular patient group, the study seeks to provide more comprehensive insights and data regarding the viability and outcomes of utilizing ultrasound guidance to perform percutaneous biopsies, potentially offering a safer and equally effective method compared to the traditional techniques used in OKT patients.

## 2. Materials and Methods

Following the approval of the ethics committee, we conducted a retrospective investigation of patients who had undergone either OKT or RAKT in our clinic between the years 2013 and 2024. In total, eighty-nine patients who had undergone ultrasound-assisted percutaneous needle biopsy due to the suspicion of renal dysfunction were included in this study.

The study aimed to comprehensively analyze various aspects related to these patients. We recorded their demographic characteristics, which provided insights into the age, gender, and other relevant background information of the participants. Additionally, the size of the biopsy samples and the number of glomeruli present in these samples were meticulously documented. These data were crucial for assessing the quality and adequacy of the biopsy procedures.

We also monitored and recorded the rates of graft loss and complications associated with the biopsy intervention. Ultrasonography and Doppler ultrasonography were conducted in patients exhibiting a decline of greater than 1 g/dL in post-biopsy blood count analyses or presenting with symptoms such as pain, hypotension, or tachycardia, with the objective of detecting potential complications associated with the biopsy procedure. The hemorrhage was defined as a new perinephritic collection around the transplanted kidney through ultrasonography.

This study was conducted in strict accordance with the ethical guidelines set forth by the Helsinki and Istanbul Declarations. Ethical approval for this research was granted by the research ethics committee of the University of Health Sciences, Bakırkoy Dr. Sadi Konuk Education and Research Hospital.

By examining these various parameters, the study sought to provide a thorough evaluation of the safety and efficacy of ultrasound-assisted percutaneous needle biopsy in the context of renal dysfunction in transplanted kidneys. The findings aimed to contribute valuable data to the existing body of knowledge and to inform future clinical practices regarding biopsy techniques in kidney transplant patients.

To reduce the potential for bias in patient selection, individuals who had undergone biopsy before and after biopsy in the RAKT group were incorporated into the OKT group.

We employ the RAKT technique described by Sood et al. [3], where the transplanted kidney is extra-peritonealized using a pre-prepared peritoneal flap over the iliopsoas muscle at the final stage of the operation. All grafts were placed in the right iliac fossa.

### 2.1. Biopsy Method

All biopsy procedures were conducted by an experienced interventional radiologist with the assistance of ultrasonography. Biopsy specimens were obtained from the upper pole of the transplanted kidney. A fully automatic biopsy needle (GEOTEK ESTACORE^®^ 16G Geotech Healthcare Products, Ankara, Turkey) was used in all patients.

### 2.2. Statistical Analysis

The Statistical Package for Social Science (IBM SPSS Statistics New York, NY, USA) version 23.0 was used for statistical analysis. Continuous variables were expressed as median with interquartile range, and categorical variables were expressed as percentages. The data (Hg decrease and day after transplantation) showed an abnormal distribution according to the Kolmogorov–Smirnov test. The significance of differences for abnormally distributed variables was determined using the Mann–Whitney U test. The data are presented as the mean and standard deviation. The variables of age, BMI, length of biopsy, and number of glomeruli demonstrated a normal distribution, thereby allowing the use of parametric tests for analysis. The independent sample *t*-test was employed for this purpose. The data are presented as the median and interquartile range (25th to 75th percentile). The Pearson Chi-Square and Fisher’s Exact Test were used for the analysis of categorical variables. Continuous variables were analysed with descriptive statistical analysis. The statistical significance level was set at *p* < 0.05.

## 3. Results

A total of sixty-four patients underwent OKT while twenty-five patients underwent RAKT. The mean age of all patients was 40.61 (±12.26) years. In terms of gender distribution, sixty of the patients were male, accounting for 67.4% of the study population, while twenty-nine were female, representing 32.6%. When comparing the two groups, there were no statistically significant differences observed in terms of age, gender, body mass index (BMI), or the presence of cadaveric versus living donors.

The analysis of biopsy samples revealed that the average number of glomeruli in the OKT group was 20.84 (±12.28), whereas in the RAKT group, it was 22.08 (±11.15). This difference was not statistically significant. Similarly, the length of biopsy samples did not show significant variation between the groups, with the OKT group having an average sample length of 2.71 (±1.08) cm and the RAKT group having an average length of 3.06 (±0.88) cm. Detailed data regarding the demographic characteristics and biopsy results are presented in Table 1.

Focusing on the OKT group, one patient experienced organ injury during the procedure. Additionally, five patients (7.8%) developed hemorrhages, six patients (9.4%) had hematuria, and eight patients (12.5%) reported new-onset pain at the biopsy site during the postoperative period. In the RAKT group, one patient (4%) developed hematuria, and five patients (20%) experienced new-onset pain at the biopsy site. All patients who encountered complications were managed with conservative treatment approaches.

The patients were divided into two groups based on the timing of their biopsy. The first group (Group 1) comprised individuals who underwent biopsy within the first seven days post-transplantation, while the second group (Group 2) comprised individuals who underwent biopsy between days seven and thirty-one post-transplantation. Complications, including bleeding, macroscopic hematuria, pain, and organ injury, were observed on the first day following biopsy in both groups. The analysis yielded no statistically significant differences in the incidence of complications between the two groups.

For those patients who experienced hemorrhage and a subsequent drop in hemoglobin levels, erythrocyte replacement therapy was administered to four individuals. Patients who reported pain were treated with paracetamol to alleviate their symptoms. No instances of arteriovenous fistula or perirenal infection were observed in any patient.

When comparing the overall complication rates between the OKT and RAKT groups, no statistically significant differences were found. The detailed classification of observed complications according to the Common Terminology Criteria for Adverse Events (CTCAE v4.0) of the U.S. National Cancer Institute is provided in Table 2 [4].

## 4. Discussion

Following the groundbreaking achievement of the first successful renal transplantation from a living donor performed by Dr. Joseph E. Murray and his team in 1954, the field of renal transplantation has seen substantial advancements. These advancements, particularly in transplantation immunology and surgical techniques, have played a crucial role in establishing renal transplantation as the standard therapy for treating end-stage renal failure [5,6]. This pioneering procedure marked the beginning of a new era in medical science, demonstrating that organ transplantation could be a viable and life-saving treatment for patients with irreversible kidney damage.

In 1968, Mathew et al. made a significant contribution to the field by describing the percutaneous renal allograft biopsy. This method became an essential tool for diagnosing and evaluating dysfunctions that develop in the allograft kidney following transplantation [7]. Despite the development of numerous non-invasive examination techniques over the years, the percutaneous renal biopsy has remained the gold standard for assessing renal dysfunction. Its ability to provide direct tissue samples from the transplanted kidney allows for a detailed histopathological evaluation, which is crucial for accurate diagnosis and management [8].

Redfield et al. reported a complication rate of 1.8% following percutaneous renal biopsy in transplanted kidneys, highlighting the procedure’s relative safety [1]. However, the literature indicates that the most common complication associated with this procedure is perinephric hematoma, with incidence rates varying widely between 0.5% and 11% [9,10]. In our own study, we found that the most frequently observed complication was pain emerging after the intervention, underscoring the need for effective pain management strategies.

A study conducted by Gilmore et al. identified specific risk factors for complications following percutaneous renal biopsy. They reported that a platelet count of less than 60,000 and an international normalized ratio (INR) value greater than 1.3 significantly increased the risk of complications [11]. These findings highlight the importance of thorough pre-procedural evaluation and optimization of coagulation parameters to minimize risks. Another study demonstrated that reducing the diameter of the biopsy needle and using automatic needles significantly decreased complication rates [12,13]. These advancements in biopsy techniques have contributed to enhancing the safety and efficacy of the procedure.

However, Redfield et al. noted that complication rates were significantly higher in patients who underwent biopsy within the first week post-transplantation [1]. They attributed this increased risk to the absence of a tamponade effect in the retroperitoneal region, which results from insufficient scar tissue development. This finding underscores the importance of timing in performing percutaneous renal biopsies and suggests that delaying the procedure beyond the first week post-transplantation might reduce the risk of complications.

In our study, we categorized patients into two groups based on the timing of their biopsy: Group 1, who had the biopsy within the first seven days post-transplantation, and Group 2, who had the biopsy after the seventh day. Our analysis revealed no statistically significant differences in complication rates between these two groups, suggesting that other factors might also play a role in determining the risk of complications.

While open surgery continues to be the standard approach for renal transplantation, the advent of robotic surgery marked a significant milestone in the field. The first RAKT was performed by Hoznek et al. in 2002, paving the way for the widespread adoption of this innovative technique [14,15,16,17,18]. The precision and minimally invasive nature of robotic surgery have made RAKT an attractive option for many medical centers around the world.

In open kidney transplantation, the extraperitoneal location of the kidney minimizes the risk of damage to other organs during percutaneous renal biopsy. However, in RAKT, where the kidney is located intraperitoneally, there is a perceived increased risk of additional organ damage, particularly to the bowel, during percutaneous biopsy. This anatomical difference necessitates a careful approach to biopsy procedures in patients who have undergone RAKT.

Despite the lack of comprehensive data on the percutaneous renal biopsy method for renal allograft dysfunction post-RAKT, Tzvetanov et al. recommended laparoscopy-assisted percutaneous renal biopsy for these patients [14]. While this method is reliable, it has disadvantages such as the need for general anesthesia and reduced patient comfort. These drawbacks highlight the need for further research to optimize biopsy techniques for RAKT patients. Tsai et al. suggested that the indication for RAKT should be restricted to patients with morbid obesity due to the increased risks associated with renal biopsy in this population [16]. Giulianotti et al. acknowledged the intraperitoneal localization as a disadvantage for biopsy but emphasized that the overall advantages of RAKT outweigh this drawback [19].

We employ the RAKT technique described by Sood et al., where the transplanted kidney is extra-peritonealized using a pre-prepared peritoneal flap over the iliopsoas muscle at the final stage of the operation [3]. This method provides a more stable kidney position, relatively isolated from intraabdominal tissues, thereby reducing the risk of additional organ damage during post-transplantation allograft biopsy. In our study, we compared the percutaneous renal biopsy results of patients who underwent RAKT with those who underwent OKT, finding no significant differences in complication rates (*p* = 0.52).

Robot-assisted kidney transplantation is preferred to open surgery in obese patients, mainly because it is associated with lower surgical complication rates and similar patient and graft survival. In our study, there was no difference in BMI between the OKT and RAKT groups. Although there were more obese patients in the RAKT group, there was no significant difference in biopsy complications between the groups in terms of BMI.

There were several limitations to this study. Firstly, as a retrospective study, there may have been a selection bias in OKT group. To reduce the potential for bias in patient selection, individuals who had undergone biopsy before and after biopsy in the RAKT group were incorporated into the OKT group. Secondly, the study had a relatively small sample size.

A notable advancement in the RAKT procedure is the extra-peritonealization of the kidney following transplantation. This surgical technique involves the repositioning of the transplanted kidney to an extraperitoneal location, which is achieved by creating a peritoneal flap over the iliopsoas muscle during the final stage of the operation. This repositioning isolates the kidney from intraabdominal tissues, reducing the risk of injury to surrounding organs during the biopsy procedure. As a result, the reliability and safety of percutaneous needle biopsies are significantly enhanced in these patients.

As the popularity of RAKT continues to grow, driven by its minimally invasive nature and improved patient outcomes, there is an increasing need for more comprehensive studies to determine the most appropriate biopsy protocols for these cases. Current data, while promising, are limited by small sample sizes and retrospective study designs. To establish robust clinical guidelines, future research should focus on larger patient cohorts and employ prospective study designs. Such studies would provide a higher level of evidence, validate current findings, and offer more definitive recommendations for clinical practice.

Moreover, these future studies should investigate various aspects of biopsy techniques, including the timing of biopsies, the impact of different needle sizes and types, and the potential role of adjunctive technologies in further enhancing biopsy safety and efficacy. By addressing these areas, researchers can contribute to the development of optimized biopsy protocols that ensure both the safety of patients and the accuracy of diagnostic evaluations.

## 5. Conclusions

Ultrasound-assisted percutaneous needle biopsy has proven to be a reliable and effective method for obtaining tissue samples in patients who have undergone robot-assisted kidney transplantation. This method is applicable in both indication-based biopsies, which are performed due to specific clinical concerns, and protocol biopsies, which are conducted at predetermined intervals as part of post-transplantation monitoring. The precision of ultrasound guidance enhances the accuracy of needle placement, thereby minimizing complications and improving diagnostic yield.

In conclusion, while ultrasound-assisted percutaneous needle biopsy is currently a reliable method for patients who have undergone RAKT, continued research is essential to refine these techniques and establish standardized protocols. The ultimate goal is to ensure that all patients receive the highest standard of care, with minimized risks and maximized diagnostic benefits.

## Figures and Tables

**Table 1:** Patient characteristics and peri- and post-procedural data.

Parameters (Mean ± SD)	Total (n = 89)	OKT (n = 64)	RAKT (n = 25)	*p*
Age (years)	40.61 (±12.26)	40.78 (±12.59)	40.16 (±11.61)	0.831 *
Gender (n; %)				0.564 ^+^
Male	60 (67.4)	42 (65.6)	18 (72)	
Female	29 (32.6)	22 (34.4)	7 (28)	
BMI (kg/m^2^)	26.41 (±2.47)	26.43 (±1.87)	26.35 (±3.65)	0.901 *
Bx 7 (n; %)	11 (12.4)	2 (8)	9 (14.1)	0.721 ^!^
Bx 31 (n; %)	38 (42.7)	29 (45.3)	9 (36)	0.425 ^+^
Cadaveric Donor (n; %)	15 (16.9)	13 (20.3)	2 (8)	0.217 ^!^
Hg Decrease (g/dL)	1.20 (1.10–1.30)	1.2 (1.10–1.37)	1 (1–)	0.170 *
>1 g/dL Hg Decrease n (%)	19 (21.3)	16 (25)	3 (12)	0.179
Day after Transplantation (median; IQR)	79 (14–344)	62.50 (13–313.75)	107 (18–400)	0.409 *
No. of glomerulus	21.19 (±11.92)	20.84 (±12.28)	22.08 (±11.15)	0.663 *
Length of biopsy (cm)	2.81 (±1.03)	2.71 (±1.08)	3.06 (±0.88)	0.160 *
Complication (n; %)	19 (21.3)	15 (23.4)	4 (16)	0.442 ^!^

* Independent *t* test; Mann–Whitney U; ^!^ Fisher exact test; ^+^ Pearson Chi square test. OKT: open kidney transplantation; RAKT: robot-assisted kidney transplantation; BMI: body mass index; Hg: hemoglobulin; Bx 7: biopsy within postoperative first 7 days; Bx 31: biopsy within postoperative 31 days; SD: standard deviation; IQR: interquartile range.

**Table 2:** Complications according to CTAE.

Complication (n; %)	OKT (n:15, 23.4%)	RAKT (n:4; 16%)	Total (n:19; 21.3%)	*p*
CTCAE Grade 1				
Macroscopic Hematuria	6 (9.4%)	1 (4%)	7 (7.9%)	0.668 *
Pain	8 (12.5%)	5 (20%)	13 (14.6%)	0.504 *
CTCAE Grade 2				
Organ Injury	1 (1.6%)	0	1 (1.1%)	1 *
Hemorrhage	5 (7.8%)	0	5 (5.6%)	0.316 *

CTCAE: Common Terminology Criteria for Adverse Events; OKT: open kidney transplantation; RAKT: robot-assisted renal transplantation. * Fisher exact test.

## Data Availability

The raw data supporting the conclusions of this article will be made available by the authors on request.
